# 4-[2-(Cyclo­hexa-1,4-dien-1-yl)eth­oxy]benzene-1,2-dicarbonitrile

**DOI:** 10.1107/S1600536811042164

**Published:** 2011-10-22

**Authors:** Zeynep Keleşoğlu, Elif Çelenk Kaya, Afşin Ahmet Kaya, Halit Kantekin, Orhan Büyükgüngör

**Affiliations:** a Department of Physics, Ondokuz Mayıs University, TR-55139 Samsun, Turkey; b Gümüşhane University, TR-29000 Gümüşhane, Turkey; c Department of Chemistry, Karadeniz Technical University, TR-61080 Trabzon, Turkey

## Abstract

In the title compound, C_16_H_14_N_2_O, the dihedral angle between the aromatic rings is 70.23 (6)°. The linking chain has a zigzag conformation. In the crystal, mol­ecules are linked by weak inter­molecular C—H⋯N hydrogen bonds, forming a zigzag chain along the *c* axis.

## Related literature

For background to the use of phthalonitriles and phthalocyanines, see: McKeown (1998 ▶); Leznoff & Lever (1989–1996 ▶); Moser & Thomas (1983 ▶). For the crystal structures of related cyclo­hexa-1,4-dienyl rings, see: Dialer *et al.* (2004 ▶); Jandacek & Simonsen (1969 ▶); Therrien & Süss-Fink (2006 ▶); Lou & Hu (2009 ▶). For further synthetic details, see: Menzek *et al.* (2008 ▶). For C N bond lengths, see: Nesi *et al.* (1998 ▶); Ocak Ískeleli *et al.* (2005 ▶); Subbiah Pandi *et al.* (2002 ▶); Yu *et al.* (2010 ▶). For the Hirshfeld Rigid-Bond test, see: Hirshfeld (1976 ▶).
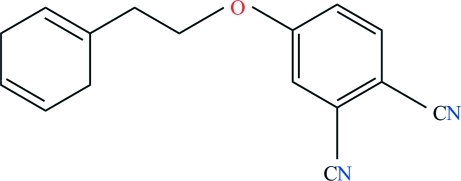


## Experimental

### 

#### Crystal data

C_16_H_14_N_2_O
*M* *_r_* = 250.29
Orthorhombic, 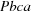

*a* = 8.6291 (3) Å
*b* = 27.5913 (14) Å
*c* = 11.7246 (5) Å
*V* = 2791.5 (2) Å^3^
*Z* = 8
Mo *K*α radiation
μ = 0.08 mm^−1^
*T* = 296 K
0.45 × 0.37 × 0.32 mm

#### Data collection

Stoe IPDS 2 diffractometer
Absorption correction: integration (*X-RED32*; Stoe & Cie, 2002 ▶) *T*_min_ = 0.968, *T*_max_ = 0.983
19074 measured reflections
2790 independent reflections
1901 reflections with *I* > 2σ(*I*)
*R*_int_ = 0.047

#### Refinement

*R*[*F* ^2^ > 2σ(*F* ^2^)] = 0.050
*wR*(*F* ^2^) = 0.139
*S* = 1.07
19074 reflections
172 parameters
2 restraints
H-atom parameters constrained
Δρ_max_ = 0.11 e Å^−3^
Δρ_min_ = −0.13 e Å^−3^

### 

Data collection: *X-AREA* (Stoe & Cie, 2002 ▶); cell refinement: *X-AREA*; data reduction: *X-RED32* (Stoe & Cie, 2002 ▶); program(s) used to solve structure: *SHELXS97* (Sheldrick, 2008 ▶); program(s) used to refine structure: *SHELXL97* (Sheldrick, 2008) ▶; molecular graphics: *ORTEP-3 for Windows* (Farrugia, 1997 ▶); software used to prepare material for publication: *WinGX* (Farrugia, 1999 ▶).

## Supplementary Material

Crystal structure: contains datablock(s) I, global. DOI: 10.1107/S1600536811042164/fk2042sup1.cif

Structure factors: contains datablock(s) I. DOI: 10.1107/S1600536811042164/fk2042Isup2.hkl

Supplementary material file. DOI: 10.1107/S1600536811042164/fk2042Isup3.cml

Additional supplementary materials:  crystallographic information; 3D view; checkCIF report

## Figures and Tables

**Table 1:** Hydrogen-bond geometry (Å, °)

*D*—H⋯*A*	*D*—H	H⋯*A*	*D*⋯*A*	*D*—H⋯*A*
C14—H14⋯N2^i^	0.93	2.56	3.453 (3)	162

Symmetry code: (i) 
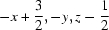
.

**Fig. 1.**
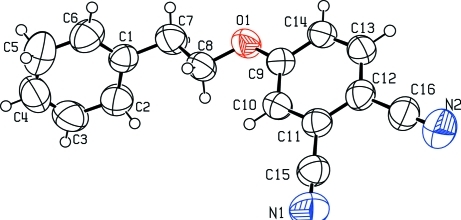
An ORTEP view of (I), with the atom-numbering scheme and 50% probability displacement ellipsoids.

**Fig. 2.**
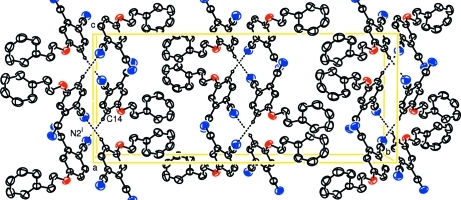
A packing diagram for (I), showing the C—H···N hydrogen bonds. H atoms not involved in hydrogen bonding (dashed lines) have been omitted for clarity.[Symmetry code; (i): 3/2 - x, -y, -1/2 + z].

## supplementary crystallographic information

### Comment

Substituted phthalonitriles are generally used for preparing symmetrically and unsymmetrically peripherally substituted phthalocyanine complexes and sub-phthalocyanines (McKeown, 1998; Leznoff & Lever, 1989–1996). Phthalocyanines were first developed as dyes and pigments (Moser & Thomas, 1983). Over the last few years, a great deal of interest has focused on the synthesis of phthalocyanine derivatives due to their applications in many fields, such as chemical sensors, electrochromic devices, batteries, semiconductive materials, liquid crystals, non-linear optics and photodynamic therapy (PDT) (Leznoff & Lever, 1989–1996).

In the title compound, C_16_H_14_N_2_O_1_, (I), both rings cyclohexa-1,4-dienyl and phthalonitrile are almost planar with r.m.s. deviations of 0.0074 Å and 0.0183 Å, respectively. They are linked by a CH_2_—CH_2_—O group with zigzag conformation and form a dihedral angle of 70.23 (6)° (Fig.1).

The C=C double bonds of the cyclohexa-1,4-dienyl ring measure 1.344 (3) Å and 1.319 (4) Å  and are similar to those in other works (Dialer *et al.*, 2004; Jandacek & Simonsen, 1969; Therrien & Süss-Fink, 2006; Lou & Hu, 2009). The C15≡N1 and C16≡N2 triple bonds, which are placed at *meta* and *para* position, are 1.141 (2) Å, and in good agreement with literature values (Subbiah Pandi *et al.*, 2002; Yu *et al.*, 2010; Nesi *et al.*, 1998; Ocak Ískeleli *et al.*, 2005).

In the crystal, molecules are linked by weak intermolecular C—H ···N hydrogen bonds, forming a zigzag chain along *c* axis (Table 1 and Fig.2).

### Experimental

The mixture of 2-(cyclohexa-1,4-dienyl)ethanol and 2-cyclohexenylethanol (0.4 g, 3.2 mmol) (Menzek *et al.*, 2008) was dissolved in dry DMF (20 ml) under N_2_ atmosphere and 4-nitrophthalonitrile (0.57 g, 3.2 mmol) was added to the solution. After stirring 10 min. finely ground anhydrous K_2_CO_3_ (2.2 g, 16 mmol) was added portion wise within 2 h with efficient stirring. The reaction mixture was stirred under N_2_ at 50 °C for 5 days. The solution was poured into ice-water (100 g) and was stirred 24 h. The mixture of solid product was filtered, washed with water and dried *in vacuo* over P_2_O_5_. The obtained product was purified from the column chromatography on silica gel with hexane-ethyl acetate (9:1) as eluents. The compound was crystallized from ethanol. Yield: 0.49 g (61%). Anal. Calcd (%) C,76.78; H,5.64; N,11.19. Found:*C*,76.38; H,5.43; N,11.16. IR (KBr tablets), ν_max_(cm^-1^): 3027 (Ar—H), 2929–2883 (Aliphatic. C—H), 2230 (C≡N), 1598, 1561, 1492, 1321, 1254, 1172, 1097, 1016, 959, 836, 749, 666. ^1^H NMR (CDCI_3_), (δ: p.p.m.): 8.61 (d, 1H, Ar—H), 7.26–7.21 (m, 2H, Ar—H), 5.69 (s, 2H, CH), 5.54 (s, 1H, CH), 4.14 (t, 2H, O—CH2), 2.68–2.64 (m, 4H, CH_2_), 2.48 (t, 2H, CH_2_). ^13^C NMR (CDCl_3_), (δ: p.p.m.): 162.29, 135.48, 130.66, 129.16, 128.92, 127.16, 124.41, 124.02, 121.80, 119.98, 119.60, 115.99, 67.95, 36.64, 29.42, 26.97. MS (ES_+_), (m/*z*): 250 [*M*]_+_.

### Refinement

All H atoms were positioned with idealized geometry using a riding model, [C—H = 0.93–0.97Å and *U*_iso_ = 1.2*U*_eq_(C)]. DELU instruction is applied to C11—C15 and C12—C16 atoms for Hirshfeld Rigid-Bond Test (Hirshfeld, 1976).

### Crystal data

**Table:**

C_16_H_14_N_2_O	*F*(000) = 1056
*M**_r_* = 250.29	*D*_x_ = 1.191 Mg m^−^^3^
Orthorhombic, *P**b**c**a*	Mo *K*α radiation, λ = 0.71073 Å
Hall symbol: -P 2ac 2ab	Cell parameters from 19074 reflections
*a* = 8.6291 (3) Å	θ = 1.5–26.2°
*b* = 27.5913 (14) Å	µ = 0.08 mm^−^^1^
*c* = 11.7246 (5) Å	*T* = 296 K
*V* = 2791.5 (2) Å^3^	Block, colorless
*Z* = 8	0.45 × 0.37 × 0.32 mm

### Data collection

**Table:**

Stoe IPDS 2 diffractometer	2790 independent reflections
Radiation source: fine-focus sealed tube	1901 reflections with *I* > 2σ(*I*)
graphite	*R*_int_ = 0.047
rotation method scans	θ_max_ = 26.2°, θ_min_ = 1.5°
Absorption correction: integration (*X-RED32*; Stoe & Cie, 2002)	*h* = −10→10
*T*_min_ = 0.968, *T*_max_ = 0.983	*k* = −34→34
19074 measured reflections	*l* = −14→14

### Refinement

**Table:**

Refinement on *F*^2^	Primary atom site location: structure-invariant direct methods
Least-squares matrix: full	Secondary atom site location: difference Fourier map
*R*[*F*^2^ > 2σ(*F*^2^)] = 0.050	Hydrogen site location: inferred from neighbouring sites
*wR*(*F*^2^) = 0.139	H-atom parameters constrained
*S* = 1.07	*w* = 1/[σ^2^(*F*_o_^2^) + (0.0714*P*)^2^ + 0.1026*P*] where *P* = (*F*_o_^2^ + 2*F*_c_^2^)/3
19074 reflections	(Δ/σ)_max_ < 0.001
172 parameters	Δρ_max_ = 0.11 e Å^−^^3^
2 restraints	Δρ_min_ = −0.13 e Å^−^^3^

### Special details

**Table:**

Geometry. All e.s.d.'s (except the e.s.d. in the dihedral angle between two l.s. planes) are estimated using the full covariance matrix. The cell e.s.d.'s are taken into account individually in the estimation of e.s.d.'s in distances, angles and torsion angles; correlations between e.s.d.'s in cell parameters are only used when they are defined by crystal symmetry. An approximate (isotropic) treatment of cell e.s.d.'s is used for estimating e.s.d.'s involving l.s. planes.
Refinement. Refinement of *F*^2^ against ALL reflections. The weighted *R*-factor *wR* and goodness of fit *S* are based on *F*^2^, conventional *R*-factors *R* are based on *F*, with *F* set to zero for negative *F*^2^. The threshold expression of *F*^2^ > σ(*F*^2^) is used only for calculating *R*-factors(gt) *etc*. and is not relevant to the choice of reflections for refinement. *R*-factors based on *F*^2^ are statistically about twice as large as those based on *F*, and *R*- factors based on ALL data will be even larger.

### Fractional atomic coordinates and isotropic or equivalent isotropic displacement parameters (Å^2^)

**Table:**

	*x*	*y*	*z*	*U*_iso_*/*U*_eq_	
C1	−0.0085 (2)	0.17483 (7)	0.35845 (15)	0.0732 (5)	
C2	−0.1054 (3)	0.17362 (9)	0.45699 (19)	0.1004 (7)	
H2A	−0.0401	0.1683	0.5232	0.120*	
H2B	−0.1738	0.1459	0.4504	0.120*	
C3	−0.2008 (3)	0.21659 (12)	0.4786 (2)	0.1193 (9)	
H3	−0.2667	0.2169	0.5413	0.143*	
C4	−0.1957 (3)	0.25488 (11)	0.4112 (3)	0.1166 (9)	
H4	−0.2589	0.2812	0.4279	0.140*	
C5	−0.0983 (4)	0.25734 (10)	0.3152 (3)	0.1248 (9)	
H5A	−0.0287	0.2846	0.3250	0.150*	
H5B	−0.1620	0.2640	0.2489	0.150*	
C6	−0.0055 (3)	0.21439 (9)	0.2917 (2)	0.1004 (7)	
H6	0.0585	0.2144	0.2278	0.120*	
C7	0.0900 (3)	0.13131 (8)	0.33115 (18)	0.0918 (6)	
H7A	0.0266	0.1023	0.3346	0.110*	
H7B	0.1298	0.1343	0.2541	0.110*	
C8	0.2225 (2)	0.12638 (8)	0.41224 (16)	0.0822 (5)	
H8A	0.2832	0.1560	0.4132	0.099*	
H8B	0.1843	0.1204	0.4888	0.099*	
C9	0.4477 (2)	0.07643 (6)	0.43332 (14)	0.0665 (4)	
C10	0.4881 (2)	0.09851 (6)	0.53531 (14)	0.0686 (4)	
H10	0.4238	0.1218	0.5675	0.082*	
C11	0.6245 (2)	0.08556 (6)	0.58867 (14)	0.0663 (4)	
C12	0.7225 (2)	0.05090 (6)	0.54140 (14)	0.0690 (4)	
C13	0.6805 (2)	0.02936 (6)	0.43832 (14)	0.0724 (5)	
H13	0.7451	0.0063	0.4053	0.087*	
C14	0.5449 (2)	0.04191 (6)	0.38548 (14)	0.0702 (5)	
H14	0.5177	0.0272	0.3170	0.084*	
C15	0.6671 (2)	0.10975 (7)	0.69314 (16)	0.0772 (5)	
C16	0.8642 (2)	0.03802 (7)	0.59768 (17)	0.0810 (5)	
N1	0.7011 (2)	0.12993 (7)	0.77426 (15)	0.1066 (6)	
N2	0.9776 (2)	0.02813 (8)	0.64194 (17)	0.1077 (6)	
O1	0.31679 (15)	0.08620 (5)	0.37450 (10)	0.0824 (4)	

### Atomic displacement parameters (Å^2^)

**Table:**

	*U*^11^	*U*^22^	*U*^33^	*U*^12^	*U*^13^	*U*^23^
C1	0.0677 (11)	0.0829 (11)	0.0690 (10)	−0.0002 (9)	−0.0071 (8)	−0.0004 (9)
C2	0.0990 (15)	0.1089 (16)	0.0931 (14)	0.0081 (13)	0.0154 (12)	0.0115 (12)
C3	0.111 (2)	0.155 (3)	0.0922 (15)	0.0361 (18)	0.0114 (14)	−0.0140 (17)
C4	0.123 (2)	0.1029 (19)	0.124 (2)	0.0322 (16)	−0.0330 (18)	−0.0331 (17)
C5	0.125 (2)	0.0928 (17)	0.156 (3)	0.0011 (15)	−0.031 (2)	0.0192 (17)
C6	0.0925 (15)	0.1142 (18)	0.0944 (14)	−0.0011 (13)	0.0070 (12)	0.0216 (13)
C7	0.0899 (14)	0.1034 (15)	0.0820 (12)	0.0126 (12)	−0.0152 (11)	−0.0191 (11)
C8	0.0801 (12)	0.0911 (13)	0.0752 (10)	0.0164 (10)	−0.0095 (9)	−0.0200 (10)
C9	0.0712 (11)	0.0671 (10)	0.0614 (9)	0.0016 (8)	0.0002 (8)	−0.0007 (7)
C10	0.0719 (11)	0.0701 (10)	0.0638 (9)	0.0034 (8)	0.0014 (8)	−0.0063 (8)
C11	0.0699 (10)	0.0668 (10)	0.0622 (8)	−0.0031 (8)	0.0018 (8)	0.0029 (7)
C12	0.0719 (10)	0.0657 (10)	0.0693 (9)	−0.0004 (8)	0.0043 (8)	0.0126 (8)
C13	0.0825 (12)	0.0655 (10)	0.0692 (10)	0.0078 (9)	0.0106 (9)	0.0038 (8)
C14	0.0858 (12)	0.0653 (10)	0.0597 (9)	0.0038 (9)	0.0046 (8)	−0.0030 (7)
C15	0.0802 (12)	0.0800 (12)	0.0714 (10)	−0.0017 (10)	−0.0091 (9)	−0.0026 (8)
C16	0.0802 (11)	0.0812 (12)	0.0816 (11)	0.0078 (10)	−0.0013 (9)	0.0094 (9)
N1	0.1198 (15)	0.1121 (14)	0.0877 (11)	−0.0017 (11)	−0.0250 (10)	−0.0168 (10)
N2	0.0960 (14)	0.1210 (15)	0.1059 (14)	0.0233 (11)	−0.0129 (11)	0.0093 (11)
O1	0.0831 (9)	0.0900 (8)	0.0740 (7)	0.0161 (7)	−0.0133 (6)	−0.0210 (6)

### Geometric parameters (Å, °)

**Table:**

C1—C6	1.344 (3)	C8—O1	1.445 (2)
C1—C2	1.426 (3)	C8—H8A	0.9700
C1—C7	1.506 (3)	C8—H8B	0.9700
C2—C3	1.465 (3)	C9—O1	1.351 (2)
C2—H2A	0.9700	C9—C10	1.386 (2)
C2—H2B	0.9700	C9—C14	1.387 (2)
C3—C4	1.319 (4)	C10—C11	1.380 (2)
C3—H3	0.9300	C10—H10	0.9300
C4—C5	1.406 (4)	C11—C12	1.392 (2)
C4—H4	0.9300	C11—C15	1.443 (3)
C5—C6	1.457 (4)	C12—C13	1.395 (2)
C5—H5A	0.9700	C12—C16	1.434 (3)
C5—H5B	0.9700	C13—C14	1.369 (3)
C6—H6	0.9300	C13—H13	0.9300
C7—C8	1.493 (3)	C14—H14	0.9300
C7—H7A	0.9700	C15—N1	1.141 (2)
C7—H7B	0.9700	C16—N2	1.141 (2)
			
C6—C1—C2	120.2 (2)	H7A—C7—H7B	107.9
C6—C1—C7	120.86 (19)	O1—C8—C7	107.84 (14)
C2—C1—C7	118.99 (18)	O1—C8—H8A	110.1
C1—C2—C3	116.8 (2)	C7—C8—H8A	110.1
C1—C2—H2A	108.1	O1—C8—H8B	110.1
C3—C2—H2A	108.1	C7—C8—H8B	110.1
C1—C2—H2B	108.1	H8A—C8—H8B	108.5
C3—C2—H2B	108.1	O1—C9—C10	124.26 (15)
H2A—C2—H2B	107.3	O1—C9—C14	115.85 (15)
C4—C3—C2	121.7 (2)	C10—C9—C14	119.89 (16)
C4—C3—H3	119.1	C11—C10—C9	119.46 (16)
C2—C3—H3	119.1	C11—C10—H10	120.3
C3—C4—C5	122.5 (2)	C9—C10—H10	120.3
C3—C4—H4	118.7	C10—C11—C12	121.04 (16)
C5—C4—H4	118.7	C10—C11—C15	118.87 (16)
C4—C5—C6	116.2 (2)	C12—C11—C15	120.06 (16)
C4—C5—H5A	108.2	C11—C12—C13	118.68 (16)
C6—C5—H5A	108.2	C11—C12—C16	120.35 (16)
C4—C5—H5B	108.2	C13—C12—C16	120.98 (17)
C6—C5—H5B	108.2	C14—C13—C12	120.43 (17)
H5A—C5—H5B	107.4	C14—C13—H13	119.8
C1—C6—C5	122.6 (2)	C12—C13—H13	119.8
C1—C6—H6	118.7	C13—C14—C9	120.50 (16)
C5—C6—H6	118.7	C13—C14—H14	119.8
C8—C7—C1	111.70 (15)	C9—C14—H14	119.8
C8—C7—H7A	109.3	N1—C15—C11	178.3 (2)
C1—C7—H7A	109.3	N2—C16—C12	179.3 (2)
C8—C7—H7B	109.3	C9—O1—C8	117.90 (13)
C1—C7—H7B	109.3		
			
C6—C1—C2—C3	1.2 (3)	C9—C10—C11—C15	178.35 (16)
C7—C1—C2—C3	−179.0 (2)	C10—C11—C12—C13	0.1 (2)
C1—C2—C3—C4	−1.0 (4)	C15—C11—C12—C13	−177.84 (16)
C2—C3—C4—C5	−0.5 (4)	C10—C11—C12—C16	179.71 (16)
C3—C4—C5—C6	1.7 (4)	C15—C11—C12—C16	1.8 (2)
C2—C1—C6—C5	0.1 (3)	C11—C12—C13—C14	−0.5 (3)
C7—C1—C6—C5	−179.7 (2)	C16—C12—C13—C14	179.92 (16)
C4—C5—C6—C1	−1.5 (4)	C12—C13—C14—C9	0.4 (3)
C6—C1—C7—C8	107.6 (2)	O1—C9—C14—C13	−179.97 (15)
C2—C1—C7—C8	−72.2 (3)	C10—C9—C14—C13	0.1 (3)
C1—C7—C8—O1	−175.64 (17)	C10—C9—O1—C8	8.2 (3)
O1—C9—C10—C11	179.61 (16)	C14—C9—O1—C8	−171.72 (16)
C14—C9—C10—C11	−0.5 (3)	C7—C8—O1—C9	177.27 (17)
C9—C10—C11—C12	0.4 (3)		

### Hydrogen-bond geometry (Å, °)

**Table:**

*D*—H···*A*	*D*—H	H···*A*	*D*···*A*	*D*—H···*A*
C14—H14···N2^i^	0.93	2.56	3.453 (3)	162.

Symmetry codes: (i) −*x*+3/2, −*y*, *z*−1/2.
